# The Gut and the Translocated Microbiomes in HIV Infection: Current Concepts and Future Avenues

**DOI:** 10.20411/pai.v9i1.693

**Published:** 2024-05-24

**Authors:** Krystelle Nganou-Makamdop, Daniel C. Douek

**Affiliations:** 1 Institute of Clinical and Molecular Virology, University Hospital Erlangen, Erlangen, Germany; 2 Human Immunology Section, Vaccine Research Center, National Institute of Allergy and Infectious Diseases, National Institutes of Health, Bethesda, Maryland

**Keywords:** HIV, Microbial translocation, Gut microbiome, Translocated microbiome, Inflammation, Immune recovery

## Abstract

It is widely acknowledged that HIV infection results in disruption of the gut's mucosal integrity partly due a profound loss of gastrointestinal CD4^+^ T cells that are targets of the virus. In addition, systemic inflammation and immune activation that drive disease pathogenesis are reduced but not normalized by antiretroviral therapy (ART). It has long been postulated that through the process of microbial translocation, the gut microbiome acts as a key driver of systemic inflammation and immune recovery in HIV infection. As such, many studies have aimed at characterizing the gut microbiota in order to unravel its influence in people with HIV and have reported an association between various bacterial taxa and inflammation. This review assesses both contra-dictory and consistent findings among several studies in order to clarify the overall mechanisms by which the gut microbiota in adults may influence immune recovery in HIV infection. Independently of the gut microbiome, observations made from analysis of microbial products in the blood provide direct insight into how the translocated microbiome may drive immune recovery. To help better understand strengths and limitations of the findings reported, this review also highlights the numerous factors that can influence microbiome studies, be they experimental methodologies, and host-intrinsic or host-extrinsic factors. Altogether, a fuller understanding of the interplay between the gut microbiome and immunity in HIV infection may contribute to preventive and therapeutic approaches.

## INTRODUCTION

The human microbiota represents the collection of microbes existing in and on the human body. These microbes are of diverse nature and include bacteria, viruses, and eukaryotes belonging to taxa such as Fungi or Apicomplexa. While some of these micro-organisms are known to be pathogenic and associated with a variety of infectious and sometimes life-threatening diseases, it is increasingly recognized that non-pathogenic organisms may have a profound influence on immunity in humans. Recent estimates have determined that humans harbor 1.3 times more bacterial cells than human cells [1] although such an estimate still does not consider non-bacterial micro-organisms. The more commonly used term microbiome encompasses the microbiota as well as the habitat it colonizes [2]. In that sense, it is well recognized that the human microbiome varies depending on the anatomical site in the human body [3]. The human gut contains about 1000 bacterial species that altogether account for at least 2 million genes which greatly outnumbers the 20,000 human genes [4]. With these characteristics, the gut undisputedly accounts for the largest microbiota of the human body and thus the largest share of non-human matter to which the host immune system may be exposed. Generally, the precise composition of microbial species at a given body site as well as optimally functioning mucosal immune cells may determine shiﬅs between health and disease states. In the field of HIV research, there is a high interest in understanding the role that distinct microbiomes play in disease pathogenesis. Given the common transmission route of HIV through sexual intercourse, studies on genital tract microbiomes are of high interest, as reviewed previously [5, 6]. In this review, we will focus on the gut microbiome and address our current knowledge of how the human gut microbiome changes during the course of HIV infection, as well as how these changes in turn influence HIV pathogenesis in treated or untreated settings.

### Methods to Measure the Gut Microbiome

To begin, it is important to highlight the various methods used to measure the gut microbiome as each method has strengths and weaknesses that may limit the degree of interpretation inferred from study findings. So far, some of the methods most commonly used to assess the gut microbiome rely on the detection of microbial nucleic acids. Targeted measurement of microbial nucleic acids entails expansion of defined genomic regions prior to sequencing and annotation of the retrieved sequences to distinguish between microbial taxonomic groups. The most common approach for targeted sequencing-based analysis of the microbiota relies on the prokaryotic 16S ribosomal RNA genes (16S rRNA) that span approximately 1500bp and hold conserved regions interspaced between 9 variable regions labeled V1 to V9 [7].

Contrary to prior assumptions that targeting variable regions of the 16S rRNA gene may be sufficient to identify taxa at the genus level or above, recent studies have shown that PCR-based techniques using universal bacterial primers to amplify distinct variable regions of the 16S rRNA gene may have biases for certain bacterial groups, leading to an overrepresentation of these bacterial groups, or can dramatically affect the numbers of taxa found [8]. Full-length sequencing would resolve the bias of targeting 16S rRNA gene subregions but would also require sequencing of long fragments. This is not widely used due its technical challenges and, until recently, incompatibility with high throughput methodologies. Sequence agnostic measurement of microbial nucleic acids covering all nucleic acids regardless of source or genomic location are commonly performed by shotgun metagenomic sequencing that has been shown to provide a broader assessment of microbial diversity as well as the number of species detected, particularly when longer sequencing reads are obtained [9]. That said, it is also important to consider that among the wide array of bioinformatics tools used for metagenomic analysis of the microbiota, some have been shown to have relatively high false discovery rates [10].

Aside from measurement of microbial nucleic acids, assessment of microbial products, typically metabolites, is also used for analysis of the microbiome. Humans rely on intestinal microbiota to metabolize complex dietary carbohydrates. During this process, degradation of dietary fibers results in the production of organic acids, gases, and short-chain fatty acids (SFCAs) [11]. SFCAs increase the secretion of antimicrobial peptides by epithelial cells, and though it is unclear if changes in SFCA levels in human diseases are a cause or consequence of the pathology, a reduced abundance of SFCA-producing bacteria has been shown in disease settings such as inflammatory bowel diseases [12, 13]. Mechanisms by which SCFAs modulate innate and adaptive immunity in viral diseases and in particular in HIV infection were recently reviewed [14]. Gas chromatography and mass spectrometry are commonly used to measure gut SCFAs and other small molecules [15], from which the gut microbial composition can be estimated.

### Immunological Markers Associated with Changes in the Gut Microbiome

Disruption of the integrity and the function of the intestinal barrier can lead to the unchecked influx of bacterial components into the circulation, thereby leading to systemic inflammation. As such, several markers measured in the blood can be indicative of functional alterations in the gut microbiome and the resulting microbial translocation.

Lipopolysaccharide (LPS), a component of the gram-negative bacterial outer membrane, can alter homeostasis in the gut by promoting local inflammation and disrupting tight junctions [16], all of which can lead to systemic inflammation. This systemic inflammation is a result of the recognition of bacterial components by immune cells such as monocytes that express CD14, a glycosylphosphatidylinositol-linked receptor for various microbial moieties including, among others, LPS [17], lipoteichoic acid (LTA [18]), and peptidoglycan (PGN [19]). Because gram-negative gut bacteria have LPS in their cell membrane whereas PGN and LTA are major cell wall components of gram-positive bacteria [20], increased blood levels of LPS indicate translocation of distinct gut bacteria. Nonetheless, these various bacterial products reaching systemic sites can all trigger immune responses, albeit via independent pathways [21], through their interaction with CD14. As a result of the interaction between monocytes and LPS or LTA, soluble CD14 (sCD14) is shed by activated monocytes [22]; in addition, plasma levels of sCD14 have been shown to independently predict mortality in HIV infection [23]. LPS-binding proteins are secreted by enterocytes in response to inflammatory stimuli and bind to LPS to trigger independent pathways that lead to the clearance of LPS [24]. Endotoxin-core antibodies (EndoCAbs) are IgM or IgG antibodies against the endotoxin core of LPS which can bind and neutralize circulating LPS.

In comparison to healthy persons, patients with sepsis were found to have lower blood levels of IgM and IgG EndoCAbs [25]. Thus, lower EndoCAbs may indicate higher LPS exposure. Aside from markers related to LPS, microbial translocation can also be identified by measurement of markers of gut barrier integrity. In a healthy setting, gut epithelial cells are held together by tight junctions that support the transport of water and electrolytes across the intestinal epithelium. The tight junction-associated protein zonulin is a regulator of epithelial and endothelial barrier function. Although upregulated expression and higher circulating levels of zonulin have been shown to associate with increased permeability of the gut epithelium in disease settings [26], recent studies refuting the specificity of commercially available assays for the measurement of zonulin due to cross-reactivity with other proteins [27, 28] warrant a cautious interpretation of data that suggests a link between gut epithelial damage and zonulin.

Finally, intestinal fatty-acid binding protein (I-FABP) is considered a marker of intestinal barrier dysfunction as it is normally present in epithelial cells of the small intestine but is released into the circulation upon damage of the gut mucosa [29]. While it is widely acknowledged that the gut shows increased permeability during HIV infection [30], whether ART can revert this damage and to what extent remains unclear. In fact, circulating levels of IFABP increase during early [31, 32] and long-term ART [33] though plasma sCD14 levels remain stable or diminish [32, 33]. This confirms that other factors aside from microbial translocation drive inflammation and immune activation in HIV infection. In addition, these observations suggest that understanding the relationship between IFABP levels and gut permeability may require consideration of additional mechanisms other than epithelial damage. For instance, a recent study suggests that next to indicating enterocyte damage, IFABP may also indicate gut maturation [34].

### The Gut Microbiota in HIV Infection

Given the paucity of data on non-bacterial communities of the gut microbiota during HIV infection, the upcoming sections of this review will cover gut bacteria. The bacterial fraction of the human gut microbiota is predominantly composed of 5 phyla: Firmicutes make up 60% to 80% of bacterial taxa followed by Bacteroidetes that account for 20% to 40%. Although at low prevalence, the phyla Verrucomicrobia, Actinobacteria, and to a lesser extent, Proteobacteria are among the top 5 bacterial phyla in the gut microbiota [35]. Studies on the gut microbiota in HIV infection have for the most part performed 16S rRNA V3-V4 gene sequencing of stool samples or rectal swabs, and aimed to clarify whether HIV infection influences microbial diversity—specifically, alpha and beta diversity. Alpha diversity is a measure of the diversity within a sample as defined by richness or evenness, whereas beta diversity determines the variability in the taxa composition among samples within a habitat. An overview of several studies on the gut microbiota during HIV infection [36–53] is presented in Table 1. While various studies have reported a lower alpha diversity in HIV infection, others have reported no effect, irrespective of ART status. Clearly, a global comparison is not enough to decipher the potential effect of HIV on the composition of the gut microbiota. Comparison of taxa distribution at the phylum level has indicated a higher abundance of Proteobacteria in infected persons irrespective of ART status [36, 37, 42], whereas others have reported a high ratio of *Prevotella* to *Bacteroides*, 2 genera of the phylum of Bacteroidetes [43, 47]. However, both the microbial diversity and phylum distribution have so far not been able to consistently explain the changes in plasma levels of immune makers linked to alterations in the gut microbiota. In addition, the absence of a consistent effect on either the microbial diversity or the phyla distribution, even when accounting for ART, indicates that comparison at lower taxonomic levels may be needed to understand how HIV infection changes the gut microbiome.

**Table 1.** Comparative Overview of Studies on the Gut Microbiota in HIV Infection

Study	Cohort	Male/Female ratio	Location	CD4 T cell count median (range or IQR ^†^)	Sample	Method	Global observations in the gut microbiota during HIV infection	Genera significantly enriched or depleted in the HIV group?	Microbiota Associated with immunological/clinical measures?	Ref.
Dillon et al.2014	18 VU, 14 HU	VU: 13/5; HU: 9/5	USA	VU: 425 (238–782) HU: 724 (468–1,071)	Colon biopsies, Stool,	16S rRNA gene V4 sequencing	Lower relative abundance of Firmicutes and higher relative abundance of Proteo-bacteria in colon biopsies but not in fecal aspirates or stool samples. No effect on Shannon diversity	Yes (VU)	Yes	[28]
Dinh et al.2015	21 ART, 16 HU	ART: 17/4 HU: 12/4	USA	ART: 668 (^†^424–870) HU: NR	Stool	16S rRNA gene V3–V5 sequencing	No difference in alpha diversity but distinct beta diversity between the groups and a higher abundance of Proteobacteria in the ART group	Yes (ART)	Yes	[29]
Dubourg et al. 2016	13 VU, 18 ART, 27 HU	VU& ART: 23/9 HU: 20/7	France	VU: 145 (2–1600) ART: 424 (43–1048) HU: NR	Stool	16S rRNA gene V3–V4 sequencing	Lower alpha diversity and higher abundance of Gammaproteobacteria in ART and VU combined compared to HU.	Yes (VU&ART combined)	Yes	[30]
Liu et al. 2019	14 ART, 22 HU	ART: 14/0 HU: 21/1	USA	ART: 570 (^†^364) HU: NR	Stool	16S rRNA gene V3–V4 sequencing	No difference in alpha diversity.	Yes (ART)	Yes	[31]
Lozu-pone et al.2013	3 VU-R, 11 VU, 6 R-ART, 8 ART, 13 HU	VU-R: 2/1 VU: ll/0 R-ART: 5/1 ART: 7/1 HU: 8/5	USA	VU-R: 614 (107–1,342) VU: 551 (270–1095) R-ART: 609 (547–681) ART: 483 (204–876) HU: NR	Stool	16S rRNA gene V4 sequencing	Higher alpha diversity in VU compared to both HU and ART groups. No difference in alpha diversity between HU and ART groups.	Yes (VU)	Soluble or cellular markers not measured.	[32]
Monaco et al. 2016	42 VU, 40 ART, 40HU	VU: 11/31, ART: 20/20, HU: 20/20	Uganda	VU: 225 (113–382) ART: 396 (283–490) HUNR	Stool	Bacteriome: 16S rRNA gene V4 sequencing Virome: Phi29 amplification of virus-like particle DNA	Among HIV subgroups, low alpha diversity in VU with CD4 T cell count <200 only. No difference in beta diversity by HIV status	Yes (VU&ART combined)	Yes	[33]
Mtuluet al.2014	21 ART, 22HU	ART: 16/5 HU: 17/5	USA	ART: 425+/-259 HU: ND	Terminalileum, right & leit colon, stool	16S rRNA gene sequencing-unspecified region	Less OTUs, lower richness and increase abundance of Gammaproteobacteria in ART	Yes (ART)	Yes	[34]
Noguera Julian et al.2016	Barcelona: 129 HIV+ (VU, ART, EC), 27 HU ------- Stockholm: 77 VU, 7 HU	Barcelona: 31 female, 124 male of which 100 MSMs Stockholm: 33 female, 51 male of which 19 MSMs	Spain, Sweden	Barcelona HIV+: 700 (462–860) Stockholm VU: 480 (380–630) HU: NR	Stool	16S rRNA gene V3–V4 sequencing	Lower diversity in HIV+ compared to HIV-MSMs but generally higher diversity in MSMs compared to non-MSMs	Unclear	Yes	[35]
Nowak et al.2015	28 VU, 3EC, 9HU	VU: 14/14 EC: 2/1 HU: 5/4	Sweden	VU: 345 (120–530) EC: 1040 (340–2470) HU: ND	Stool	16S rRNA gene V3–V4 sequencing	Lower alpha diversity in HIV+ irrespective of EC inclusion, with lowest number of species among persons with lowest CD4 T cell count. ART start lowered number of species and Shannon index whereas beta diversity increased	Yes (VU)	Yes	[36]
Nowak et al.2017	41 VU 34 ART, 55HU	All MSM	Nigeria	NR	Rectal swabs	16S rRNA gene V3–V4 sequencing	No difference in alpha diversity between groups. Within the phylum of Bac-teroidetes, the ART group had lower species richness and evenness	Yes (ART)	ND	[37]
Perez Santiago et al. 2013	13 VU (follow-up under ART)	All MSM	USA	520 (91–971)	Rectal swabs	16S rRNA gene V6 sequencing	Diversity data not reported. Depleted Bacteroidales and enriched Lactobacillales and Enterobacteriales in 6 of 11 participants	ND	Yes	[38]
Pinto Cardoso et al. 2017	33 ART, 10HU	HU: 6/4 ART-EFV: 16/2 ART-PL: 13/2	Mexico	ART - EFV: 457 (†276–564.5) ART - PI: 559 (†369–703) HU: 995 (†856.5–1238)	Stool	16S rRNA gene sequencing V3–V4	Lower alpha diversity in ART irrespective of treatment regimen High Prevotalla/Bacteroides ratio in HU whereas some individuals within the ART group were either Prevotella dominant or Bacteroides dominant	Yes (ART)	Yes	[39]
Rhoades et al. 2019	58 ART, 47 HU	All male	USA	ART: 689 HU: 1198	Rectal swabs	16S rRNA gene V4 sequencing	No difference in alpha or beta diversity.	Yes (ART)	Yes	[40]
Serrano-Villar et al. 2017	42 ART	All MSM	Spain	605 (†475–819)	Rectal biopsies and stool	16S rRNA gene sequencing V3–V4	Higher alpha diversity in rectal mucosa compared with feces	Yes (ART)	ND	[41]
Serrano-Villar et al. 2017	12 VU, 8 ART-INR, 15 ART-IR, 9 HU	VU: 11/1 ART-INR: 8/0 ART-IR: 13/2 HU: 6/3	Spain	IR: 561 (426–794) INR: 291 (230–324) VU: 558 (432–646) HU: 762 (653–878)	Stool	16S rRNA gene sequencing - unspecified region	Alpha diversity was the highest in VU and lowest in INR	Yes (VU & ART)	Yes	[42]
Vujkovic-Cvijin et al. 2013	6 VU, 18 ART, 1 LTNP, 9 HU	All male	USA	VU: 356.3 (313–819) ART: 374.5 (251 1110) LTNP: 505 HU: 803 (460–1487)	Rec-to-sig-moid biopsies	16S rRNA gene microarray	No significant differences between ART and HU in community evenness or richness	Yes (VU)	Yes	[43]
Vujkovic-Cvijin et al. 2020	80 ART, 80 HU	ART: 61/19 (incl. 44 MSM) HU: 60/20 (incl.39 MSM)	Netherlands	ART: Female-760 (635, 895) MSM - 620 (427.5, 750) Non-MSM 600 (450, 800) HU: NR	Stool	16S rRNA gene V4 sequencing	Lower alpha diversity in ART presumably due to predominant depletion of Clostridiales members of the Lachnospiraceae and Ruminococcaceae families. Alpha diversity lower in ART compared to HU irrespective of sexual practice	Yes (ART)	Yes	[44]
Zhao et al 2022 J Med Virol	56 ART-IR, 41 ART - INR, 51 HU	All MSMs	China	HU: NR ART-IR: 528 (440–600) ART-INR: 194 (126–239)	Stool	16S rRNA gene V3–V4 sequencing	Higher alpha diversity in ART groups compared to HU with no distinction between IR and INR but all 3 groups showed distinct beta diversity. Higher relative abundance of Proteobacteria and lower relative abundance of Act-inobacteria in ART groups compared to HU.	Yes (ART)	Yes	[45]

Detailed lists of bacterial genera significantly enriched or depleted in these studies as well as associations with immunological measures are presented in Supplementary Table 1.

Abbreviations: ART: ART-treated HIV infection; HU: HIV uninfected; INR: immune non-responders; IR: immune responders; LTNP: HIV-infected long-term non-progressor; MSM: Men who have sex with men; NA: not applicable; ND: not described; R-ART: HIV infection with recent ART initiation; VU: viremic untreated; VU-R: viremic untreated with recent HIV infection.

### Genus-level Alterations of the Gut Microbiome in HIV Infection

The various studies presented in Table 1 have all reported the enrichment or depletion of distinct bacterial genera in HIV infection, regardless of whether the overall diversity was affected. These studies also demonstrate that different genera from the same phylum can be enriched in either HIV-infected or healthy control groups, supporting the need of analysis at low taxonomic level. Several studies cited in Table 1 have reported associations between certain bacterial genera and clinical and immunological outcome measures, thereby indicating a contribution of such genera to the mechanisms by which the gut microbiome influences the course of HIV infection. For example, the genus *Faecalibacterium* (Firmicutes phylum), was reported to be depleted in treated HIV infection compared to healthy persons and inversely correlated with plasma sCD14 [38, 42, 50] and IFABP [47].

In contrast, the family of Enterobacteriaceae (Proteobacteria phylum) enriched in treated HIV infection, was shown to positively correlate with plasma levels of sCD14 [37]. While such observations may indicate that defined bacterial genera of the gut microbiome play distinct roles in the modulation of immunity, the fact remains that even at the genus-level, reported findings are oﬅen inconsistent. For instance, in contrast to the prior studies, Monaco et al reported a positive association between the abundance of *Faecalibacterium* and plasma sCD14 levels [41], but the combination of treated and untreated infection in the analysis could have influenced this association. Importantly, different studies tended to highlight different sets of genera. Given the functional overlap that can be expected between closely related species, it is possible that distinct functional groups of bacteria, rather than unique genera, may explain the dynamics of the gut microbiome in HIV infection despite variation in species composition.

**Figure 1.**
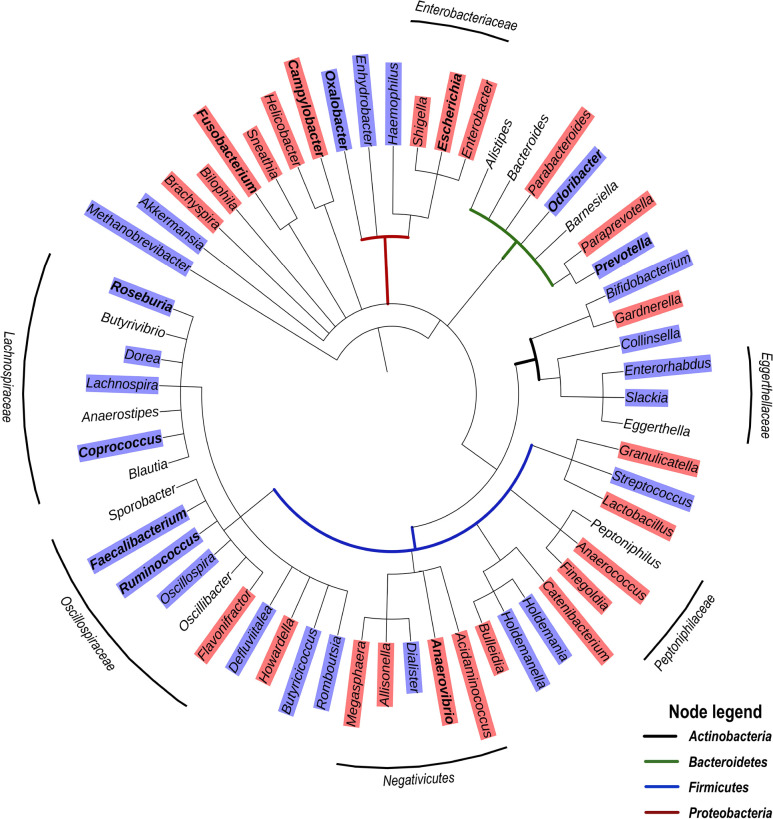
**Phylogenetic tree based on the NCBI taxonomy of genera significantly enriched (red) or depleted (blue) in ART-treated HIV infection as reported by** [37, 39, 42, 45, 47, 48, 50, 52, 53]. Genera marked in bold were reported in 2 or more studies. Genera without color shade were reported as significantly different, but the relationships varied depending on the study. Tree generated with PhyloT v2.

Based on the studies presented in Table 1, we have compiled a list of genera significantly discriminating between HIV and control groups, with most of these studies reporting genera enriched or depleted in ART-treated HIV infection. Figure 1 presents a grouping of these genera as a phylogenetic tree and provides an overview of clusters that may be generally enriched or depleted in ART-treated HIV infection. Among Firmicutes, the genera of the family of Lachnospiraceae (class of clostridia) tend to be depleted. Interestingly, the Lachnospiraceae family, known to be rich in SFCA producers [54], include species such as the probiotic *Clostridium butyricum* that promote an anti-inflammatory milieu through TGF-β signaling in dendritic cells, which prevents an increase in Enterobacteriaceae [55]. Moreover, Lachnospiraceae have been shown to degrade lyso-glycerophospholipids that inhibit CD8 T-cell activity, resulting in the maintenance of CD8 T-cell responses in the colon [56]. Another family among Firmicutes, namely the Ruminococcaceae, are also SFCA producers and include *Faecalibacterium*, an important butyrate producer in the human gut that has anti-inflammatory effects and induces the novel immunoregulatory T-cell subset CD4^+^CD8[.alpha]^+^ [57]. Of note, butyrate was shown to reduce epithelial permeability by the regulation of tight junction proteins [58]. Another cluster of bacteria depleted in treated HIV-infection belong to the Eggerthellaceae family, currently considered candidates for next-generation probiotics, and have been found to have anti-inflammatory and antioxidative properties leading to upregulation of tight junction proteins [59]. Thus, the specific reduction in abundance of Lachnospiraceae, *Faecalibacterium*, and Eggerthellaceae in HIV infection may contribute to poor control of pro-inflammatory bacteria at the gut mucosal interface. In contrast to bacterial taxa that tend to be depleted in treated HIV-infection, Figure 1 also shows an enrichment in Enterobacteriaceae (Gammaproteobacteria) as well as the Firmicutes, Negativicutes, and the Peptoniphilaceae genera *Peptoniphilus*, *Anaeorococcus,* and *Finegoldia*.

**Figure 2.**
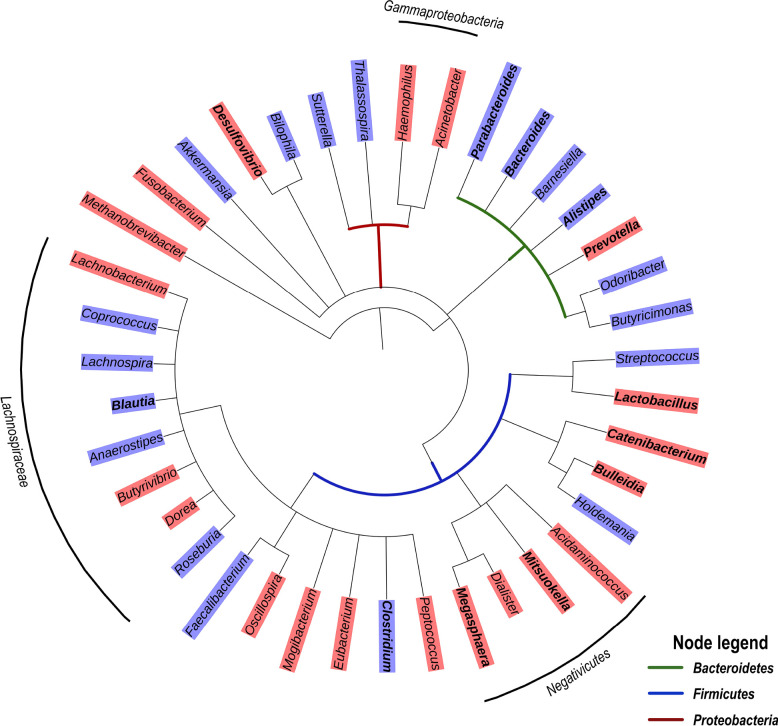
**Phylogenetic tree based on the NCBI taxonomy of genera significantly enriched (red) or depleted (blue) in untreated HIV infection as reported by** [36, 40, 44, 50]. Genera marked in bold were reported in 2 or more studies. Genera without color shade were reported as significantly different, but the relationships varied depending on the study. Tree generated with PhyloT v2.

Of note, a recent study reporting a significant enrichment of the genera *Peptoniphilus*, *Anaeorococcus,* and *Finegoldia* during community-acquired pneumonia has highlighted an association between these genera and high serum levels of C-reactive protein (CRP) and IL-6 [60]. With respect to Gammaproteobacteria that are typically associated with intestinal inflammation, it has been proposed that increased oxygen availability offers a selective advantage to facultative anaerobic bacteria such as Enterobacteriaceae, thereby driving their expansion in the gut [61]. However, increased oxygenation is likely only a partial explanation for the enrichment of these Proteobacteria taxa. For one, Gammaproteobacteria exclusively carry the gene encoding for the hexa-acetylated form of LPS that is the most potent TLR-4 agonist by 2 orders of magnitude [62]. In addition, Negativicutes, that are enriched in treated HIV-infection (Figure 1), are unique among Firmicutes in that they possess an outer membrane containing LPS [63], and were reported to have a large number of genes involved in cell envelope biogenesis that are similar to those of Gammaproteobacteria from which they were likely laterally acquired [64]. This raises the question whether Negativicutes may, similarly to Gammaproteobacteria, possess a distinct LPS triggering high pro-inflammatory responses. Altogether, these observations suggest a common enrichment of gut bacterial genera with strong pro-inflammatory capability along with the loss of anti-inflammatory genera in ART-treated HIV infection. Similar to ART-treated HIV infection, Gammaproteobacteria and Negativicutes tend to be enriched in untreated HIV infection, whereas several genera of the Lachnospiraceae family are depleted (Figure 2).

### Changes in the Gut Microbiome During HIV Infection: Cause, Consequence, or Coincidence?

As described above, several studies have reported that the gut microbiota of persons living with HIV is different from that of heathy controls. However, it is important to note that whether HIV infection directly induces dysbiosis of the human gut microbiome remains controversial. It is evident that the composition of the human microbiota varies tremendously from person to person and that dysbiosis of the gut microbiome can be induced by multiple factors such as the use of antibiotics. Importantly, current prophylactic guidelines of HIV treatment recommend administration of the antibiotics trimethoprim-sulfamethoxazole, azithromycin, or clarithromycin to persons with a CD4 T-cell count below 200/[.mu]L or 50/[.mu]L respectively [65]. Antibiotic use is oﬅen controlled for in studies on the gut microbiota in HIV infection by excluding persons who had received antibiotics in the 1 to 3 months prior to the study. However, it has been shown that several commonly used antibiotics including Azithromycin allow for selection of resistant taxa, altering the gut microbiota that is still not fully restored aﬅer 6 months [66]. As such, whether dysbiosis is caused by HIV infection or simply reflects a change in microbial composition that coincides with HIV infection but is caused by other factors remains complicated to assess in cross-sectional studies. An overwhelming majority of longitudinal studies on infection with the simian-immunodeficiency virus (SIV) in non-human primates (reviewed by [67]) have demonstrated that acute or chronic SIV infection did not significantly change the intestinal bacterial composition. While it may be argued that the SIV model may not fully recapitulate HIV-induced changes in humans, one must acknowledge that a direct effect of HIV infection on the composition of the gut micro-biome irrespective of lifestyle or antibiotic use has yet to be formally proven.

### Focus on the Translocated Microbiome

Notwithstanding the origin of the changes observed in gut microbiome during HIV infection, the idea that the gut microbiota could drive systemic inflammation due to the distinct species that are able to activate innate and adaptive immune cells has been supported by *in vitro* studies [40, 68, 69]. However, whether the systemic inflammation is a cause or consequence of alteration in the gut microbiome as well as underlying mechanisms was long unclear. Recently, we set out to clarify the relationship between the translocated microbiome and systemic inflammation in treated HIV infection. During the first 2 years aﬅer ART initiation, we observed fluctuations in plasma concentrations of a cluster of cytokines—namely IL-6, IL-1 β, IL-8, MIP-1 β—known to trigger pro-inflammatory pathways and identified these as major mediators of inflammation in our Ugandan cohort [70]. Analysis of plasma microbial fragments by shotgun metagenomic sequencing demonstrated a predominance of Proteobacteria that is in stark contrast with their low prevalence in the gut microbiota and is particularly interesting in the light of non-human primate studies showing a disproportionate translocation of Proteobacteria [71, 72]. Specifically, we found that the Enterobacteriaceae *Serratia* were enriched in the plasma and that the ratio of *Serratia* to other bacteria positively correlated with plasma levels of IL-6, IL-1 β, IL-8, and MIP-1 β [70].

In contrast, the abundance of various genera of the phyla Actinobacteria, Proteobacteria, and Firmicutes, among which *Corynebacterium, Pseudomonas*, *Lactobacillus,* and Lachnospiraceae, inversely correlated with the plasma concentrations of these cytokines. Transcriptome analysis of sorted peripheral blood monocytes, dendritic cells (DCs), and T cells revealed gene signatures such as increased type I/II IFN responses, TNF signaling via NF-kB, and IL-6 signaling at time-points of peak inflammation and high *Serratia* ratio. Concomitantly, lower expression of genes driving Th1 and Th2 differentiation aligned with low plasma concentrations of Th1/Th2 cytokines that were associated with a low *Serratia* ratio. *In vitro* culture experiments with various species belonging to genera of the translocated microbiota confirmed an innate cytokine profile aﬅer stimulation with *Serratia* that was consistent with the *ex vivo* measured pro-inflammatory profile.

Importantly, changes in the diversity of the translocated microbiota driven by the *Serratia* ratio were associated with CD4 T-cell recovery. This important link between the translocated microbiome and clinical outcome was also confirmed in 3 independent cohorts in our study. In a separate study comparing blood bacterial profiles of HIV-uninfected persons to that of HIV-infected persons before and 48 weeks aﬅer ART, Serrano-villar et al observed that the number of species was increased before and aﬅer ART, whereas the Shannon diversity was only increased before ART [73]. Fitting our observation of high Proteobacteria abundance in the plasma of HIV-infected Ugandans [70], Serrano-villar et al found that the translocated microbiota of HIV-infected and HIV-uninfected persons as assessed by 16S rRNA gene sequencing was dominated by Gammaproteobacteria and particularly Enterobacteriaceae families [73]. While these taxa were absent in several participants 48 weeks aﬅer ART, the baseline and fold changes in Actinobacteria, the Lactobacillales order (Firmicutes), the Corynebacteriaceae (Actinobacteria) family, and the Moraxellaceae family (Proteobacteria) were significantly inversely correlated with various measures of inflammation and immune activation. In a separate study, Merlini et al compared the translocated microbiota of HIV-uninfected persons to that of HIV-infected persons before and 12 months aﬅer ART and found that the composition of the translocated microbiota as determined by 16S rRNA gene sequencing was not substantially changed by ART and that Enterobacteriales were the most detectable across individuals [74]. Comparison between immune responders and non-immune responders revealed a higher baseline prevalence of *Lactobacillus* and *Pseudomonas* in immune responders, suggesting a link between translocated bacteria and immune recovery under ART. Altogether, the aforementioned studies on the translocated microbiome in HIV infection reveal an enrichment of various Gammaproteobacteria, specifically Enterobacteriales before ART and a beneficial effect of among others Lactobacillales but not Enterobacteriales on immune recovery. As stated above, the potency of LPS derived from Gammaproteobacteria to activate innate immune cells may at least in part explain the deleterious effect of Gammaproteobacteria among the translocated microbiota in HIV infection. Whether taxa such as *Lactobacillus* also act through specific molecules they produce remain to be clarified.

Most recently, shotgun metagenomic sequencing of the translocated microbiota of HIV-uninfected or HIV-infected persons at various stages of disease did not observe a predominance of Gammaproteobacteria in the blood but rather the Bacteroidetes *Porphyromonas gingivalis* followed by the Betaproteobacteria *Burkholderia multivorans* [75]. This further emphasizes the pressing need for analytical approaches that may help navigate through inter-person and inter-study differences and reveal the overarching principles of how the gut and the translocated microbiome influence immunity in HIV infection. Another equally important point is the ability to filter out potential technical bias due to laboratory contaminants, that may especially affect samples with low microbial biomass such as blood samples [76]. Importantly, Guo et al also observed an association between bacterial taxa in the blood and inflammation-related proteins in the plasma [75].

### Unravelling the Functional Microbiome

Findings from studies on the gut microbiome in HIV infection can be integrated into a more simplified concept that a loss of anti-inflammatory bacteria and an expansion of those with pro-inflammatory properties together could contribute to systemic inflammation in HIV infection. While this establishes a clear relationship between the gut microbiome and immune recovery in HIV infection, it is far from presenting the complete picture. Several studies cited in Table 1 have reported an association between markers of inflammation and the abundance of bacterial genera that are not significantly different between HIV groups and healthy controls [36, 37, 39, 47, 53]. Thus, aside from their overall abundance as assessed by the quantification of their genomic material, other characteristics of gut microbes are likely to determine their effect on immunity. Metaproteomics (extensively reviewed by [15]) provides a complementary view of the microbiome which entails liquid chromatography-mass spectrometry analysis of peptide mixtures derived from protein extracts. Mapping of the resulting spectra to defined databases allows quantitative identification of protein identities. Such an approach allows functional analysis of the microbiome, where protein orthologs can be clustered irrespective of which microbes they are derived from.

In addition, the construction of protein-protein interaction networks and the analysis of metabolic pathways using databases such as KEGG pathways cumulatively allow the establishment of functional profiles of the microbiome. Li and colleagues have defined functional redundancy as “the ability of multiple taxonomically distinct organisms to contribute in similar ways to an ecosystem through having redundant functional traits” [77]. A convincing argument for functional redundancy is the observation from the Human Microbiome Project that despite tremendous variation in the microbial composition, functional profiles among the body sites remain stable [3]. Therefore, functional analysis of the gut microbiome represents not simply a minor addition but rather a necessary step to breakdown its complexity into findings that are consistent and biologically meaningful. Evidently, combining metagenomics (ie, what are the genes detected?), metatranscriptomics (ie, how is the expression regulated?), metaproteomics (what are the functions of the resulting proteins?) and metabolomics (what microbial small molecules are acting agents of the effect of the microbiome on immunity?) may be a powerful approach to obtain a high-resolution view into the effect of the gut microbiome on human diseases.

### Factors Influencing the Gut Microbiome

It is important to acknowledge the various factors that influence and shape the gut microbiome, as these may provide contextual nuances that should be taken into consideration for translational approaches such as development of preventive or therapeutic strategies. Studies on the gut micro-biome in HIV infection have shown that the overall diversity [44, 52] and abundance of defined taxa [36, 41, 46, 51] are associated with CD4 T-cell counts. In fact, Monaco et al concluded that the CD4 T-cell count is the most influential factor contributing to bacterial community structure, where a CD4 T-cell count under 200/[.mu]L was associated with an enrichment of Enterobacteriaceae, Ruminococcaceae*,* and Clostridiaceae [41]. Moreover, studies addressing the impact of sexual practices have reported that the gut microbiome of men who have sex with men (MSM) is different than that of non MSM-male or female; and MSM exhibited a unique microbial profile regardless of HIV or ART status [52, 78].

Irrespective of sexual practices, the nadir CD4 T-cell count was again shown to be the strongest predictor of microbial dysbiosis in the gut, with a notable increase in dysbiosis index once the CD4 T-cell count dropped below 200/[.mu]L [52]. With current therapy guidelines that aim to treat as soon as a diagnosis is made, many individuals may have initiated cART well before reaching low CD4 T-cell counts. Nonetheless, it is important to point out that many studies have assessed the gut microbiome in persons with CD4 T-cell counts well above 200/[.mu]L (Table 1) and still reported changes in the composition of the gut microbiome. Aside from antibiotic use and sexual practices, accumulating evidence has established that the gut microbiome is influenced by a plethora of factors including age [79], diet [80], sex hormones [81], lifestyle [4], ethnicity [82], geography [83], and host polymorphism [84]. Perhaps the most challenging aspect of microbiome research in the context of HIV infection resides in the ability to establish how the microbiota influences HIV pathogenesis and immunity independently of these other factors. This conundrum is certainly not limited to the field of HIV and extends to many other areas of research such as numerous infectious diseases [85], vaccinology [86], and cancer [87] that have determined an involvement of the human microbiome in respective settings. A recent metanalysis of the gut microbiome in cancer and autoimmune disease which included 37 autoimmunity and 45 cancer studies, compiling 4208 healthy human controls and 5957 disease cases from 27 countries, was able to identify 214 distinct genus-level associations with either disease, but 131 of these genera were only reported in a single study [88]. The overall lack of consistency between studies as well as microbiome features that showed opposite associations between the diseases underlines the limitation of analysis at taxonomic levels in understanding the role of the microbiome in human disease. Despite these challenges, key mechanisms of how the gut and tumor microbiomes influence human cancer through, among others, TLR-mediated cytokine signaling have inspired numerous approaches such as the administration of microbial metabolites or synthetically engineered bacteria to modulate the gut microbiome in cancer [89].

## CONCLUSION

Studies on the gut and translocated microbiomes in HIV infection suggest that translocated microbial constituents influence the systemic inflammation that is characteristic of the disease. As challenging as microbiome research may be, an important consideration for future avenues would be to employ analytical tools that decipher the functional microbiome and facilitate translation to evidence-based interventions. Thus far, limited understanding of mechanisms have likely contributed to the inefficacy of broad and untargeted interventions such as the administration of prebiotics or probiotics [90]. Moving forward, advancement towards successful interventions will likely require the use of multi-omic approaches covering the many facets of the interaction between gut microbes and immune cells, and it should aim to identify key processes that are not easily disturbed by factors such as diet or sex. Clearly, such complex studies would only be feasible in small proof-of-concept clinical trials but will be instrumental for both selection of the most promising intervention and the identification of key clinical outcome measures to include in larger clinical trials. The currently observed associations between translocated microbiota and immune recovery support the idea that microbiome-based strategies may contribute to the clinical management of HIV infection.

## SUPPLEMENTARY DATA

Supplementary materials are available at the *Pathogens and Immunity* website. Supplementary data may be provided by the authors to benefit the reader. Supplementary data are not copyedited and are the sole responsibility of the authors. Questions or comments related to supplementary materials should be addressed to the corresponding author.
