# Supplementary material for: The Gut and the Translocated Microbiomes in HIV Infection: Current Concepts and Future Avenues
Source: Pathog Immun. 2024 May 24;9(1):168–94. doi: 10.20411/pai.v9i1.693 (PMC11132393; doi:10.20411/pai.v9i1.693)
Supplement: Supplementary Table 1 [file pai-9-168-s01.pdf]

PATHOGENS AND IMMUNITY
IN ASSOCIATION WITH CASE WESTERN RESERVE UNIVERSITY AND THE RICHARD J. FASENMYER FOUNDATION

Supplemental Table 1. Overview of Significantly Enriched and Depleted Genera and Associations with Outcome Measures

Study	Cohort	Genera significantly enriched or depleted in the HIV groups	Association with immunological or clinical outcome measures	Ref.
Dillon et al. 2014	18 VU, 14 HU	Enriched in VU: *Acinetobacter* (P), *Prevotella* (B)<br><br>Depleted in VU: *Alistipes* (B), *Bacteroides* (B), *Barnesiella* (B), *Blautia* (F), *Coprococcus* (F), *Thalassospira* (P)	Relative abundance of *Prevotella* associated with various measures of T cell activation; relative abundance of *Bacteroides* associated with high plasma LTA and low CD4 T cell counts	[28]
Dihn et al. 2015	21 ART, 16 HU	Enriched in ART: *Barnesiella* (B)<br><br>Depleted in ART: *Alistipes* (B)	Abundance of *Barnesiella* correlated with plasma TNF and the family of *Enterobacteriaceae* correlated with plasma sCD14, IL1β and IFNγ	[29]
Dubourg et al. 2016	13VU, 18 ART, 27 HU	Enriched in VU& ART: *Citrobacter* (P), *Enterococcus* (P)<br><br>Depleted in VU& ART: *Bifidobacterium* (A), *Blautia* (F), *Faecalibacterium* (F) *Ruminococcus* (F), *Subdoligranulum* (F)	Enriched genera correlated with plasma CXCL9; sCD163 or sCD27. Depleted genera inversely correlated with plasma CXCL-10, sCD14, sCD27 or sCD30.	[30]
Liu et al. 2019	14 ART, 22 HU	Enriched in ART: *Anaerovibrio* (F), Allisonella (F), *Enterobacter* (P), Howardella (F), Paraprevotella (B)<br><br>Depleted in ART: *Barnesiella* (B), *Eggerthella* (A), *Odoribacter* (B), *Oscillospira* (F), *Oxalobacter* (P)	sCD163, sCD14, sTNFR2 significantly higher in HIV+ART but association only reported with genera not significantly enriched in HIV+ART.	[31]
Lozupone et al 2013	3 VU-R, 11 VU, 6 R-ART, 8 ART, 13 HU	Enriched in VU: *Catenibacterium* (F), *Bulleidia* (F), *Dialister* (F), *Oscillospira* (F), *Mitsuokella* (F), *Peptococcus* (F), *Megasphaera* (F), *Dorea* (F), *Desulfovibrio* (P), *Prevotella* (B), *Methanobrevibacter*<br><br>Depleted in VU: *Bacteroides* (B), *Parabacteroides* (B), *Alistipes* (B), *Akkermansia*, *Clostridium* (F), *Bilophila* (P),	Soluble or cellular markers not measured.	[32]
Monaco et al. 2016	42 VU, 40 ART, 40 HU	*Shigella* (P) and *Escherichia* (P) enriched in advanced HIV disease.<br><br>Highest abundance of *Adenoviridae* and *Anelloviridae* in VU&ART with CD4 < 200	Several genera of the families of *Enterobacteriaceae* (P), *Ruminococcaceae* (F) and *Clostridiaceae* (F) associated with CD4 T cell count.<br><br>93 OTUs including *Ruminococcus* (F), *Coprococcus* (F), *Blautia* (B) and *Prevotella* (B) inversely correlated with plasma sCD14.<br><br>51 taxa including *Faecalibacterium* (F), *Bacteroides* (B) and *Prevotella* (B) positively correlated with plasma sCD14.	[33]
Mtulu et al. 2014	21 ART, 22 HU	Enriched in ART: *Brachyspira*, *Campylobacter*, *Catenibacterium* (F), *Escherichia* (P)	Serum IL-6, TNF, LTA and sCD14 did not correlate with bacterial taxa enriched in the HIV groups, but correlated with several bacterial taxa depleted in the HIV group (e.g. inverse correlation between *Faecalibacterium* and sCD14)	[34]

		Depleted in ART: *Akkermansia*, *Bacteroides* (B), *Blautia* (F), *Coprococcus* (F), *Dialister* (F), *Dorea* (F), *Faecalibacterium* (F), *Lachnospira* (F), *Roseburia* (F), *Ruminococcus* (F), *Odoribacter* (B), *Oscillospira* (F)		
Noguera Julian et al. 2016	Barcelona: 129 HIV+ (VU, ART; EC), 27 HU<br>---------<br>Stockholm: 77 VU, 7 HU	Genus *Bacteroides* dominant among non-MSMs where as *Prevotella* was dominant among MSMs. Multiple genera correlating with *Prevotella* were negatively correlated with *Bacteroides* and vice versa.	*Prevotella* or *Bacteroides* clusters based on MSM status did not associate with plasma levels of sCD14, LBP, IL-6, CRP or IP-10	[35]
Nowak et al. 2015	28 VU,<br>3 EC,<br>9 HU	Enriched in VU: *Lactobacillus* (F)<br>Depleted in VU: *Faecalibacterium* (F), *Lachnobacterium* (F), *Hemophilus* (P).<br><br>Significant reduction of *Lachnospira* (F), *Oribacterium* (F), *Oscillospira* (F), *Prevotella* (B) and *Sutterella* (P) post-ART.	Among VU, the Shannon alpha diversity and the number of species each positively correlated with CD4 T cell count and CD4/CD8 ratio. The number of species inversely correlated with plasma levels of sCD14, sCD163, LPS and LBP.<br><br>No association reported between microbial genera and plasma measures.	[36]
Nowak et al. 2017	41 VU,<br>34 ART,<br>55 HU	Enriched in ART: *Peptoniphilus* (F), *Finegoldia* (F), *Anaerococcus* (F) *Campilobacter* (P)<br>Depleted in ART: *Prevotella* (B)<br><br>No difference between VU and HU.	Soluble or cellular measured of inflammation or immune activation not measured.	[37]
Perez Santiago et al. 2013	13 VU (follow-up under ART)	Analysis at genus-level not reported	Before ART, low *Lactobacillales* proportions associated with low blood CD4%, CD4/CD8 ratio, plasma sCD14, gut CD8 Ki67+, and high VL but not with plasma LPS or activated T cells (HLA-DR+ or CD38+).<br><br>After ART, the relationships between *Lactobacillales* and T cell counts were again observed at week 24 but not at week 48	[38]
Pinto Cardoso et al. 2017	33 ART,<br>10 HU	Depleted in ART: *Faecalibacterium* (F)	The genera *Roseburia* (F) and *Faecalibacterium* (F) inversely correlated with plasma IFABP levels	[39]
Rhoades et al. 2019	58 ART,<br>47 HU	Enriched in ART: *Gardnerella (A)*, *Snethia*, *Fusobacterium*, *Lactobacillus* (F), *Helicobacter* (P)<br><br>Depleted in ART: *Oxalobacter* (P), *Streptococcus* (F), *Enhydrobacter* (P), *Eggerthella* (A), *Clostridiaceae 02d06* (F)	*Prevotella* correlated with low CD4 count in ART but not HU groups	[40]
Serrano-Villar et al. 2017	42 ART	*Prevotella* (B) was the most abundant genus in mucosa and feces	Soluble or cellular measured of inflammation or immune activation not measured.	[41]

Serrano-Villar et al. 2017	12 VU, 8 ART-INR, 15 ART-IR, 9 HU	Enriched in VU: *Acidaminococcus* (F), *Bulleidia* (F), *Butyrivibrio*, *Catenibacterium* (F), *Desulfovibrio*, *Eubacterium* (F), *Fusobacterium*, *Lactobacillus* (F), *Megasphaera* (F), *Mogibacterium* (F), *Mitsuokella* (F), *Prevotella* (B),<br><br>Depleted in VU: *Alistipes* (B), *Anaerostipes* (F), *Bacteroides* (B), *Blautia* (F), *Butyricimonas* (B), *Clostridium* (F), *Holdemania* (F), *Lachnospira* (F), *Odoribacter* (B), *Parabacteroides* (B), *Roseburia* (F), *Streptococcus* (F), *Sutterella* (P)<br><br>Enriched in ART: *Acidaminococcus* (F), *Anaerovibrio* (F), *Bulleidia (F), Butyrivibrio (F), Granulicatella* (F), *Fusobacterium, Megasphaera*<br><br>Depleted in ART: *Anaerostipes* (F), *Coprococcus* (F), *Holdemania* (F), *Faecalibacterium* (F), *Odoribacter* (B), *Ruminococcus* (F)	High abundance of *Faecalibacterium* associated with high butyrate levels, which in turn correlated with low CRP and sCD14<br><br>Low *Bacteroides*, high *Prevotella* or high *Acidaminococcus* associated with high T cell activation	[42]
Vujkovic-Cvijin et al. 2013	6 VU, 18 ART, 1 LTNP, 9 HU	579 taxa were enriched (mostly *Proteobacteria*; particularly *Enterobacteriaceae* family) and 45 taxa (e.g. *Clostridia* and *Bacteroidia*) were depleted in VU compared with HU	Various measures of systemic inflammation and immune activation, CD4 count, HIV DNA or RNA as well as gut T cell responses, IDO mRNA and Kyn/Trp differentially correlated with bacteria enriched in VU or HU.	[43]
Vujkovic-Cvijin et al. 2020	80 ART, 80 HU	240 depleted taxa and 82 enriched in ART<br>Top depleted genera in ART:<br>*Slackia* (A), *Holdemanella* (F), *Clostridium* (F), *Faecalibacterium* (F), *Prevotella* (B).<br><br>Top enriched genera included *Eggerthella* (A), *Bilophila* (P), *Escherichia/Shigella* (P)	Higher Shannon diversity associated with lower plasma sCD14 levels and higher pre-ART CD4 count	[44]
Zhao et al. 2022	56 ART – IR, 41 ART – INR, 51 HU	Enriched in ART: *Escherichia* (P)<br><br>Depleted in ART. *Faecalibacterium* (B), *Bifidobacterium* (A), *Collinsella* (A), *Oscillospira* (F), *Roseburia* (F)	Various plasma markers measured (incl. sCD14) but relationship with microbial genera not reported.	[45]

Abbreviations: INR: immune non-responders; IR: immune responders; LTNP : HIV-infected long-term non-progressor; VU: viremic untreated, VU-R: viremic untreated but recent HIV infection; ART: ART-treated HIV infection; R-ART: HIV infection with recent ART initiation; HU: HIV uninfected; MSM: Men who have sex with men; NA : not applicable; ND: not described . (*) genera reported as significantly enriched of depleted are presented along with distinctions of the following phyla: A = Actinobacteria, B = Bacteroidetes, F = Firmicutes, P = Proteobacteria.
